# Endoscopic-assisted versus open fronto-orbital distraction for unicoronal craniosynostosis: morphometric and technique considerations

**DOI:** 10.1007/s00381-024-06662-8

**Published:** 2024-12-18

**Authors:** Meagan Wu, Connor S. Wagner, Dillan F. Villavisanis, Jinggang J. Ng, Benjamin B. Massenburg, Dominic J. Romeo, Gregory G. Heuer, Scott P. Bartlett, Jordan W. Swanson, Jesse A. Taylor

**Affiliations:** 1 Division of Plastic, Reconstructive, and Oral Surgery, Children’s Hospital of Philadelphia, Philadelphia, PA USA; 2 Division of Neurosurgery, Children’s Hospital of Philadelphia, Philadelphia, PA USA

**Keywords:** Fronto-orbital distraction osteogenesis, Unilateral coronal synostosis, Endoscopic, Minimally invasive

## Abstract

**Introduction:**

In an effort to maximize benefit and minimize morbidity when performing fronto-orbital distraction osteogenesis (FODO) for unilateral coronal synostosis (UCS), we have transitioned to an endoscopic-assisted approach (“endo-FODO”). This study compares photogrammetric outcomes of patients who underwent FODO via an endoscopic-assisted versus open approach.

**Methods:**

We retrospectively reviewed patients treated for UCS from 2013 to 2023. Photogrammetric outcomes at one to three years postoperatively were compared between patients who underwent endo-FODO and age- and sex-matched controls who underwent open FODO. Differences between pre- and postoperative periorbital symmetry ratios, canthal tilt symmetry, and orbital dystopia angle (ODA) were calculated.

**Results:**

Twenty patients (ten per group) underwent surgery at a mean age of 6.1 ± 1.8 and 5.4 ± 1.1 months (*p* = 0.426) and were photographed at 1.6 ± 0.9 and 1.8 ± 0.9 years (*p* = 0.597) postoperatively in the endo-FODO and open FODO groups, respectively. Patients who underwent endo-FODO demonstrated significant improvements in margin-reflex distance 1 (MRD1) symmetry ratio (*p* = 0.004), palpebral height symmetry ratio (*p* = 0.004), canthal tilt symmetry (*p* = 0.020), and ODA (*p* = 0.009). Patients who underwent open FODO likewise demonstrated significant improvements in MRD1 symmetry ratio (*p* = 0.004), palpebral height symmetry ratio (*p* = 0.033), and ODA (*p* = 0.004). All postoperative measurements as well as degrees of improvement were similar between groups (*p* > 0.05).

**Conclusions:**

Endo- and open FODO were associated with significant and comparable improvements in soft tissue periorbital symmetry and orbital dystopia at nearly two years postoperatively. While continued follow-up until cranial maturity is needed to assess the durability of aesthetic results, these data support a minimally invasive, endoscopic alternative to fronto-orbital distraction.

## Introduction

The complex craniofacial dysmorphology in unilateral coronal synostosis (UCS) represents a problem in growth vector rather than growth restriction, where asymmetric skeletal distortion leads to shortening of the anterior cranial base and anterosuperior malpositioning of the sphenoid bone [1]. Patients classically present with ipsilateral frontal and parietal flattening, supraorbital and temporal retrusion, and vertical orbital dystopia with palpebral fissure widening [2–4]. Contralateral frontal bossing and ipsilateral nasal root deviation occur in severe cases [5]. Functionally, translation of the altered orbital anatomy to the surrounding soft tissues attachments imparts an increased risk of strabismus and amblyogenic anisometropia. [4,6–8]

A broad range of methods for UCS correction exists. The conventional treatment is fronto-orbital advancement and remodeling (FOAR); however, progressive relapse and persistence of fronto-temporal retrusion [9–13] have precipitated surgical modifications over time [14–16]. Patients may alternatively undergo endoscopic strip craniectomy which has been shown to limit blood loss and hospital stay, [17–20], although the extensive duration of postoperative helmet therapy can be burdensome. Popularized by Choi et al.,[21] fronto-orbital distraction osteogenesis (FODO) has become a promising treatment modality that allows for a larger, more gradual, and less devascularizing vault expansion at an earlier age [22,23]. Among the few centers that have adopted FODO for UCS correction, studies have demonstrated a favorable safety profile [24,25] and greater correction of skull base angulation [26–28] compared to FOAR. Still, FODO as it is most commonly practiced relies on a coronal incision, which is a source of blood loss and appearance-related concerns among parents.

Leveraging the advantages of both distraction osteogenesis and less invasive, endoscopic-assisted modalities, our institution has increasingly performed FODO via a minimal-incision, endoscopic-assisted approach (“endo-FODO”) since 2019 to further mitigate perioperative morbidity and scalp scar burden [29,30]. As the morphometric changes after endo-FODO have not been characterized, we aim to compare the photogrammetric outcomes of patients who underwent FODO via an endoscopic-assisted versus open approach. Importantly, endo-FODO provides adequate access to perform all necessary osteotomies for effective fronto-orbital advancement, and so we hypothesized that this technique would achieve similar correction of unicoronal retrusion and periorbital asymmetry as our open approach.

## Methods

### Study population

Following Institutional Review Board approval at the Children’s Hospital of Philadelphia, we retrospectively reviewed patients treated for UCS from 2013 to 2023 by two pediatric craniofacial surgeons (J.A.T. and J.W.S.). Patients who underwent endo-FODO were compared to age- and sex-matched controls who underwent open FODO. We typically perform fronto-orbital distraction between four and seven months of age, following computed tomography (CT) evaluation to identify cranial deformities and to determine the osteotomy line and vector of distraction.

Demographic and surgical data were collected, including age at time of distractor application and removal. Ophthalmic characteristics were also recorded. Preoperative CT scans were collected to evaluate baseline craniometrics, and standardized clinical photographs were taken preoperatively and between one and three years postoperatively.

### Surgical technique

**Fig. 1:**
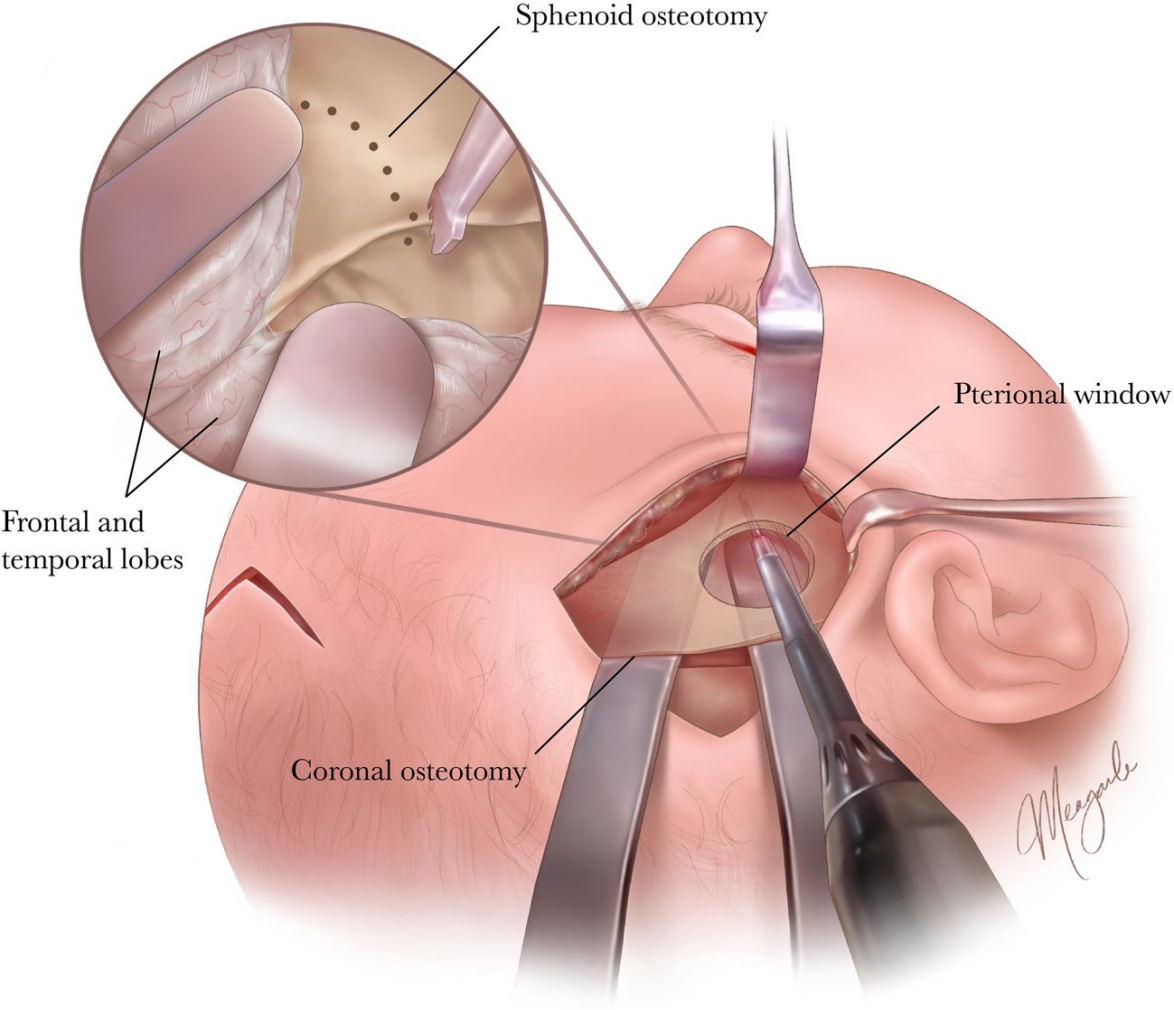
Illustration of our minimally invasive, endoscopic-assisted approach to fronto-orbital distraction osteogenesis, which demonstrates a critical view through the pterional window that serves as the predominant working portal for the sphenoid wing and orbital roof osteotomies (Copyright © 2024 Meagan Wu)

In our endo-FODO approach, exposure is obtained through three small incisions: an anterior fontanelle, ipsilateral pterion, and ipsilateral upper lateral blepharoplasty incision **(**Fig. 1**)**. [31] Through the pterional incision, the posterior half of the superficial surface of the temporalis muscle is released from the galea, and the entire deep surface is released from the periosteum to allow for rotation-advancement of the muscle flap with the transport segment during distraction [30]. With an endoscope passed through the anterior fontanelle incision, a limited coronal suturectomy is performed and a 2-cm pterional window is created just anterior to the suturectomy to serve as a working port for the sphenoid and periorbital osteotomies **(**Fig. 1**)**. These osteotomies, performed from lateral to medial, are extended to the contralateral nasofrontal junction via the blepharoplasty incision.

**Fig. 2:**
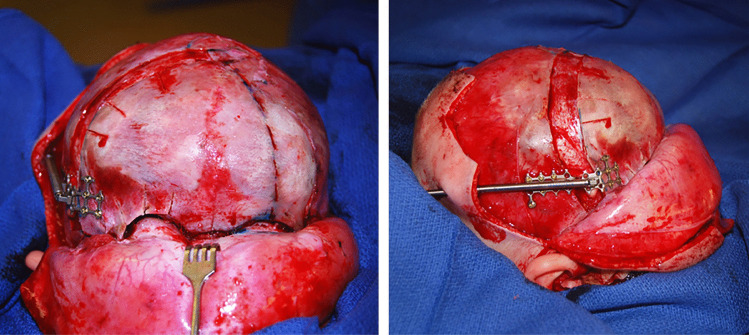
Intraoperative photographs of a female patient with right unicoronal synostosis undergoing internal distractor placement for open fronto-orbital distraction osteogenesis via a coronal incision

In our open FODO approach, via a traditional coronal incision, osteotomies typical of a unilateral FOAR extending to the medial third of the contralateral orbit are performed. [32,33] A coronal suturectomy is performed and burr hole placed at the base of the fused suture to create a pterional window for the sphenoid wing and orbital roof osteotomies. Care is taken to protect the intracranial and orbital contents. A contralateral vertical perforating osteotomy is also performed at the inflection point of the frontal deformity. Importantly, epidural dissection is restricted to the osteotomies to minimize devascularization of the frontal bone. In both approaches, cranial bone graft is placed along the suturectomy prior to closure, and a linear distractor is fixated across the coronal suturectomy with an anterior-inferior vector **(**Fig. 2**)**. Distraction begins on postoperative day two at 1 mm/day and continues until overcorrection of the orbital deformity is achieved.

### Craniometric analysis

Preoperative fine-cut head CT scans were analyzed to compare baseline morphometrics and to stratify severity at presentation. Two parameters were used to quantify facial symmetry in the frontal view based on previously published methods [28,34,35]. Orbital dystopia angle (ODA) was measured by subtracting from 90° the angle on the synostosed side formed by a vertical line connecting the nasion and anterior nasal spine (ANS) and a transverse line connecting the supraorbital notches [35], with an ODA of zero indicating perfect symmetry. Midface angulation was measured as the angle formed by the same vertical line connecting the nasion and ANS and a second line perpendicular to a transverse line connecting the bilateral orbitale. [28,34]

Orbital height was measured as the distance from the supraorbital notch to the zygomaticomaxillary suture on the inferior orbital rim, and orbital width was measured as the distance from the frontozygomatic suture to the dacryon [34]. Ratios were established to determine symmetry between orbits. In the lateral view, frontal bossing angle was measured between the sella-nasion line and a line connecting the nasion to the most anterior portion of the frontal bone (B point) on the nonsynostosed side. Skull base twist or anterior cranial fossa deviation was analyzed on Mimics Version 23.0 (Materialise, Leuven, Belgium). An axial plane was established based on the sella and bilateral orbitale, along which skull base twist was measured as the angle formed by a line connecting the sella and nasion and a line connecting the sella and opisthion. [34]

### Photogrammetric analysis

**Fig. 3:**
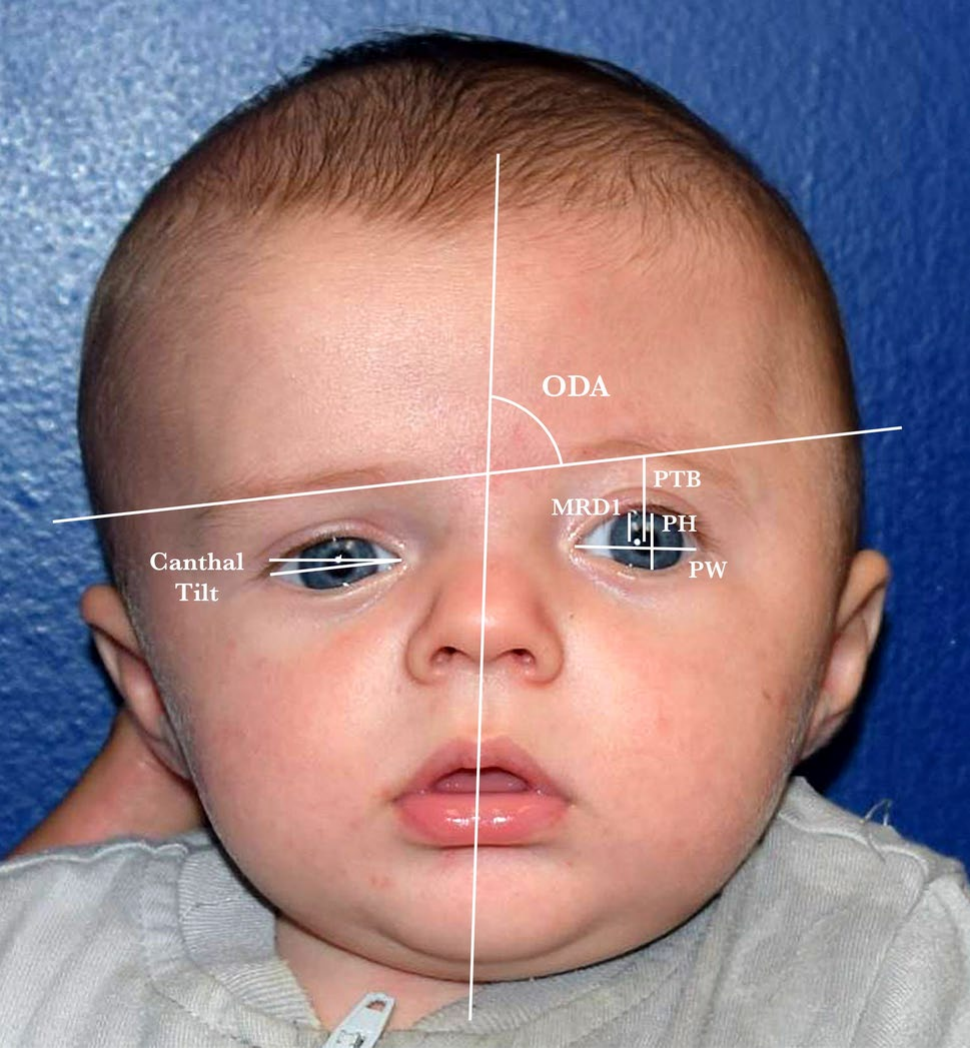
All soft tissue parameters are demonstrated on the preoperative frontal photograph of a male patient with left unicoronal synostosis who underwent fronto-orbital distraction osteogenesis at five months of age. Periorbital measurements included margin-reflex-distance 1, pupil-to-brow distance, palpebral fissure height and width, and canthal tilt angle. MRD1 margin-reflex-distance 1, PTB pupil-to-brow distance, PH palpebral height, PW palpebral width, ODA orbital dystopia angle

ImageJ photo processing software (U.S. National Institutes of Health, Bethesda, MD) was used to measure soft tissue anatomy in pixels on frontal photographs **(**Fig. 3**)**. All measurements were performed twice and averaged by one evaluator (M.W.). Linear distances included margin-reflex distance 1 (MRD1), pupil-to-brow distance (PTB), and palpebral fissure height and width. Canthal tilt was measured as the angle between a line passing through the medial and lateral canthi and a line passing through the medial canthi [36]. Canthal tilt was deemed negative if the axis was below the horizontal axis created by the medial canthi and positive if above. Soft-tissue ODA was measured by subtracting from 90° the angle on the synostosed side formed by a transverse line connecting the lower brow edges and a line intersecting the soft-tissue nasion and subnasale **(**Fig. 3**)**, [26] with zero indicating perfect symmetry.

The formula $symmetry\,\,ratio = -\left[ |(1-\left(\frac{s}{n}\right)|\times 100\right]$ was used to compare linear periorbital dimensions of the synostosed and nonsynostosed sides, with zero indicating perfect symmetry and a more negative value indicating greater asymmetry [37]. Canthal tilt symmetry was represented by $\Delta canthal\,\,tilt=|(s-n)|$. [37] Differences between pre- and postoperative symmetry ratios, canthal tilt symmetry, and soft-tissue ODA were calculated so that positive differences indicated improvement postoperatively. [37]

### Statistical analysis

Data were analyzed using JASP (Version 0.17.1; JASP Team, 2023). Outcomes were compared between treatment groups using the chi-square test for categorical variables and Mann–Whitney *U* test for continuous variables. Preoperative, postoperative, and change in photogrammetric measurements were compared within the endo- and open-FODO groups using the Wilcoxon signed-rank test. Statistical significance was set at *p* < 0.05.

## Results

Twenty patients (ten per group) had preoperative CT scans as well as pre- and postoperative clinical photographs available. There were eight females and two males in each group. Patients underwent surgery at an average age of 6.1 ± 1.8 and 5.4 ± 1.1 months in the endo- and open FODO groups, respectively **(***p* = 0.426, Table 1). Duration of distraction was similar in the endo- and open FODO groups (72.8 ± 22.6 vs. 73.4 ± 26.1 days, *p* = 0.790). Ophthalmic characteristics did not differ significantly between patients who underwent endo- and open FODO (Table 2), though fewer patients in the endo-FODO group required postoperative strabismus surgery (0% vs. 20%, *p* = 0.136).

**Table 1:** Demographic characteristics of patients with unicoronal synostosis who underwent endo- versus open fronto-orbital distraction osteogenesis

	Endo-FODO (*n* = 10)	Open FODO (*n* = 10)	*p*
Gender (%) Male Female	2 (20) 8 (80)	2 (20) 8 (80)	1.000
Race (%) White Other	8 (80) 2 (20)	9 (90) 1 (10)	0.531
Age at surgery, months	6.1 ± 1.8	5.4 ± 1.1	0.426
Duration of distraction, days	72.8 ± 22.6	73.4 ± 26.1	0.790
Laterality (%) Left Right	4 (40) 6 (60)	6 (60) 4 (40)	0.371
Photo timepoints, years Time from preop to surgery Time from surgery to postop	0.2 ± 0.2 1.6 ± 0.9	0.2 ± 0.1 1.8 ± 0.9	0.570 0.597

FODO fronto-orbital distraction osteogenesis

**Table 2:** Ophthalmic characteristics in patients with unicoronal synostosis who underwent endo- versus open fronto-orbital distraction osteogenesis (*n* = 20)

	**Endo-FODO (***n*** = 10)**	**Open FODO (***n*** = 10)**	***p***
Preoperative ophthalmopathy (%) Strabismus Trochlear nerve palsy	1 (10) 0 (0)	1 (10) 1 (10)	1.000 0.305
Postoperative ophthalmopathy (%) Strabismus New-onset postoperative strabismus Trochlear nerve palsy New-onset trochlear nerve palsy	2 (20) 1 (10) 0 (0) 1 (10)	3 (30) 2 (20) 2 (20) 1 (10)	0.606 0.531 0.136 1.000
Postoperative strabismus surgery	0 (0)	2 (20)	0.136

FODO fronto-orbital distraction osteogenesis

### Preoperative craniometrics

All baseline parameters were similar between treatment groups **(**Table 3**)**, demonstrating comparable preoperative craniofacial dysmorphology. Mean ODA was 10.8° ± 4.2° and 10.5° ± 4.1° (*p* = 0.789), midface angulation was 8.2° ± 1.8° and 6.9° ± 2.9° (*p* = 0.247), and skull base twist was 7.7° ± 4.7° and 9.2° ± 6.4° (*p* = 0.684) in the endo- and open FODO groups, respectively. Mean orbital height ratio was 1.1 ± 0.1 in the endo-FODO group and 1.2 ± 0.1 in the open FODO group (*p* = 0.481), while mean orbital width ratio was 0.9 ± 0.1 in both groups (*p* = 0.912). Mean frontal bossing angle was 114.8° ± 7.3° and 116.7° ± 11.3° (*p* = 0.579, Table 3) in the endo- and open FODO groups, respectively.

**Table 3:** Preoperative craniometrics in patients with unicoronal synostosis who underwent endo- versus open fronto-orbital distraction osteogenesis (*n* = 20)

	Endo-FODO (*n* = 10)	Open FODO (*n* = 10)	*p*
Orbital dystopia angle, °	10.76 ± 4.17	10.47 ± 4.11	0.739
Midface angulation, °	8.21 ± 1.78	6.89 ± 2.93	0.247
Skull base twist, °	7.70 ± 4.67	9.24 ± 6.35	0.684
Orbital height ratio	1.14 ± 0.08	1.16 ± 0.06	0.481
Orbital width ratio	0.90 ± 0.07	0.90 ± 0.06	0.912
Frontal bossing angle, °	114.76 ± 7.27	116.67 ± 11.26	0.579

FODO fronto-orbital distraction osteogenesis

### Photogrammetric outcomes

Postoperative photographs were taken at an average of 1.6 ± 0.9 and 1.8 ± 0.9 years postoperatively in the endo- and open FODO groups, respectively (*p* = 0.597). Preoperative soft tissue measurements were similar except for greater palpebral width asymmetry in the endo-FODO group (− 11.9 ± 6.4 vs. − 5.8 ± 4.5, *p* = 0.019). Patients who underwent endo-FODO demonstrated significant improvements in mean MRD1 symmetry ratio (− 65.0 ± 86.1 to − 12.7 ± 15.1, *p* = 0.004), palpebral height symmetry ratio (− 11.9 ± 6.4 to − 5.8 ± 5.7, *p* = 0.004), canthal tilt symmetry (5.1° ± 3.2° to 1.2° ± 1.2°, *p* = 0.020), and soft-tissue ODA (8.3° ± 2.1° to 3.9° ± 1.9°, *p* = 0.009; Table 4). Patients who underwent open FODO likewise demonstrated significant improvements in mean MRD1 symmetry ratio (− 36.0 ± 28.9 to − 6.6 ± 6.9, *p* = 0.004), palpebral height symmetry ratio (− 19.8° ± 15.4° vs. − 5.3° ± 4.1°, *p* = 0.033), and soft-tissue ODA (7.4° ± 1.7° to 3.6° ± 1.7°, *p* = 0.004; Table 4). All postoperative values as well as degrees of improvement were similar between the endo- and open FODO groups (*p* > 0.05, Table 5), with no significant correlation between soft tissue changes and age at surgery.

**Table 4:** Pre- versus postoperative morphometrics in patients with unicoronal synostosis who underwent endo- and open fronto-orbital distraction osteogenesis (*n* = 20)

	**Preoperative**	**Postoperative**	***p***
*Endo-FODO (n* = *10)*			
MRD1 symmetry ratio	− 65.0 ± 86.1	− 12.7 ± 15.1	*0.004
PTB symmetry ratio	− 23.7 ± 15.3	− 20.2 ± 14.9	0.625
Palpebral height symmetry ratio	− 47.0 ± 61.5	− 5.8 ± 5.7	*0.004
Palpebral width symmetry ratio	− 11.9 ± 6.4	− 5.3 ± 4.6	0.084
Canthal tilt symmetry, °	5.1 ± 3.2	1.2 ± 1.2	*0.020
Soft-tissue ODA, °	8.3 ± 2.1	4.9 ± 1.9	*0.009
*Open FODO (n* = *10)*			
MRD1 symmetry ratio	− 36.0 ± 28.9	− 6.6 ± 6.9	*0.004
PTB symmetry ratio	− 18.6 ± 11.4	− 10.7 ± 13.1	0.193
Palpebral height symmetry ratio	− 19.8 ± 15.4	− 5.3 ± 4.1	*0.033
Palpebral width symmetry ratio	− 5.8 ± 4.5	− 5.7 ± 3.6	0.846
Canthal tilt symmetry, °	5.1 ± 4.3	2.2 ± 2.1	0.064
Soft-tissue ODA, °	7.4 ± 1.7	3.6 ± 1.7	*0.004

*Bold *p*-values indicate statistical significance

*FODO* fronto-orbital distraction osteogenesis, *MRD1* margin-reflex distance 1, *PTB* pupil-to-brow distance, *ODA* orbital dystopia angle

**Table 5:** Comparison of morphometric outcomes in patients with unicoronal synostosis who underwent endo- versus open FODO (*n* = 20)

	**Endo-FODO** **(***n*** = 10)**	**Open FODO** **(***n*** = 10)**	***p***
MRD1 symmetry ratio Preoperative Postoperative Improvement	− 65.0 ± 86.1 − 12.7 ± 15.1 52.2 ± 73.7	− 36.0 ± 28.9 − 6.6 ± 6.9 29.5 ± 27.2	0.571 0.354 0.579
PTB symmetry ratio Preoperative Postoperative Improvement	− 23.7 ± 15.3 − 20.2 ± 14.9 3.5 ± 18.4	− 18.6 ± 11.4 − 10.7 ± 13.1 7.8 ± 18.3	0.579 0.105 0.579
Palpebral height symmetry ratio Preoperative Postoperative Improvement	− 47.0 ± 61.5 − 5.8 ± 5.7 41.2 ± 61.6	− 19.8 ± 15.4 − 5.3 ± 4.1 14.6 ± 16.9	0.140 0.820 0.218
Palpebral width symmetry ratio Preoperative Postoperative Improvement	− 11.9 ± 6.4 − 5.3 ± 4.6 6.6 ± 9.3	− 5.8 ± 4.5 − 5.7 ± 3.6 0.1 ± 6.8	*0.019 0.684 0.123
Canthal tilt symmetry, ° Preoperative Postoperative Improvement	5.1 ± 3.2 1.2 ± 1.2 3.8 ± 3.4	5.1 ± 4.3 2.2 ± 2.1 2.9 ± 5.0	0.796 0.288 0.481
Soft-tissue ODA, ° Preoperative Postoperative Improvement	8.3 ± 2.1 3.9 ± 1.9 4.4 ± 2.7	7.4 ± 2.7 3.6 ± 1.7 3.8 ± 2.9	0.218 0.853 1.000

*Bold* p-*valuesindicate statistical significance

*FODO* fronto-orbital distraction osteogenesis, *MRD1* margin-reflex distance 1, *PTB* pupil-to-brow distance, *ODA* orbital dystopia angle

## Discussion

The craniofacial differences produced by UCS present an aesthetic challenge, as the asymmetric craniofacial dysmorphology extends beyond the fronto-orbital region. Most recently, our group found that in comparison to open FODO, endo-FODO was associated with reduced anesthesia time (210 vs. 243 min, *p* = 0.029) and blood transfusion requirements (11.6 mL/kg vs. 20.3 mL/kg, *p* = 0.012) without a difference in operative duration (104 vs. 114 min, *p* = 0.607) or length of stay (3.0 days in both groups, *p* = 0.678) [38], demonstrating the feasibility and safety of a minimal-incision approach to fronto-orbital distraction for UCS correction. However, the effects of the dysmorphic bony foundation translating to the overlying soft tissues have not been well described, and it is important to confirm that the aesthetic benefits of FODO are comparable between our two approaches, especially as we transition towards endo-FODO in an effort to mitigate perioperative morbidity and scarring. Indeed, it is the altered facial symmetry and soft tissue ratios that are perceived by families and inform the need for secondary surgical interventions.

Several key findings emerge upon analyzing the short-term photogrammetric outcomes of endo- and open FODO. First, both endo- and open FODO were associated with significant improvements in periorbital soft tissue morphometrics as quantified by MRD1, palpebral height, and soft-tissue ODA, with endo-FODO additionally improving canthal tilt symmetry. Second, there were no significant differences between the degrees of postoperative soft tissue improvements between treatment groups. These results suggest the noninferiority of a minimally invasive approach to fronto-orbital distraction, which can achieve satisfactory fronto-orbital correction despite decreased surgical exposure.

### Morphometric outcomes

We first assessed three key measures of craniofacial twist preoperatively. Orbital dystopia, midface angulation, and skull base angulation were all similar between the endo- and open FODO groups, suggesting a comparable range of severity in dysmorphology at baseline. The greater degrees of orbital dystopia compared to midface angulation in both groups may reflect the differential facial asymmetry translating inferiorly from the orbits to the nasomaxillary region, justifying the common use of nasal root deviation as a surrogate for greater phenotypic severity [10,39]. It is also important that ophthalmic characteristics did not differ significantly between groups, with a particularly low incidence of new-onset postoperative strabismus and trochlear nerve palsy after endo-FODO.

**Fig. 4:**
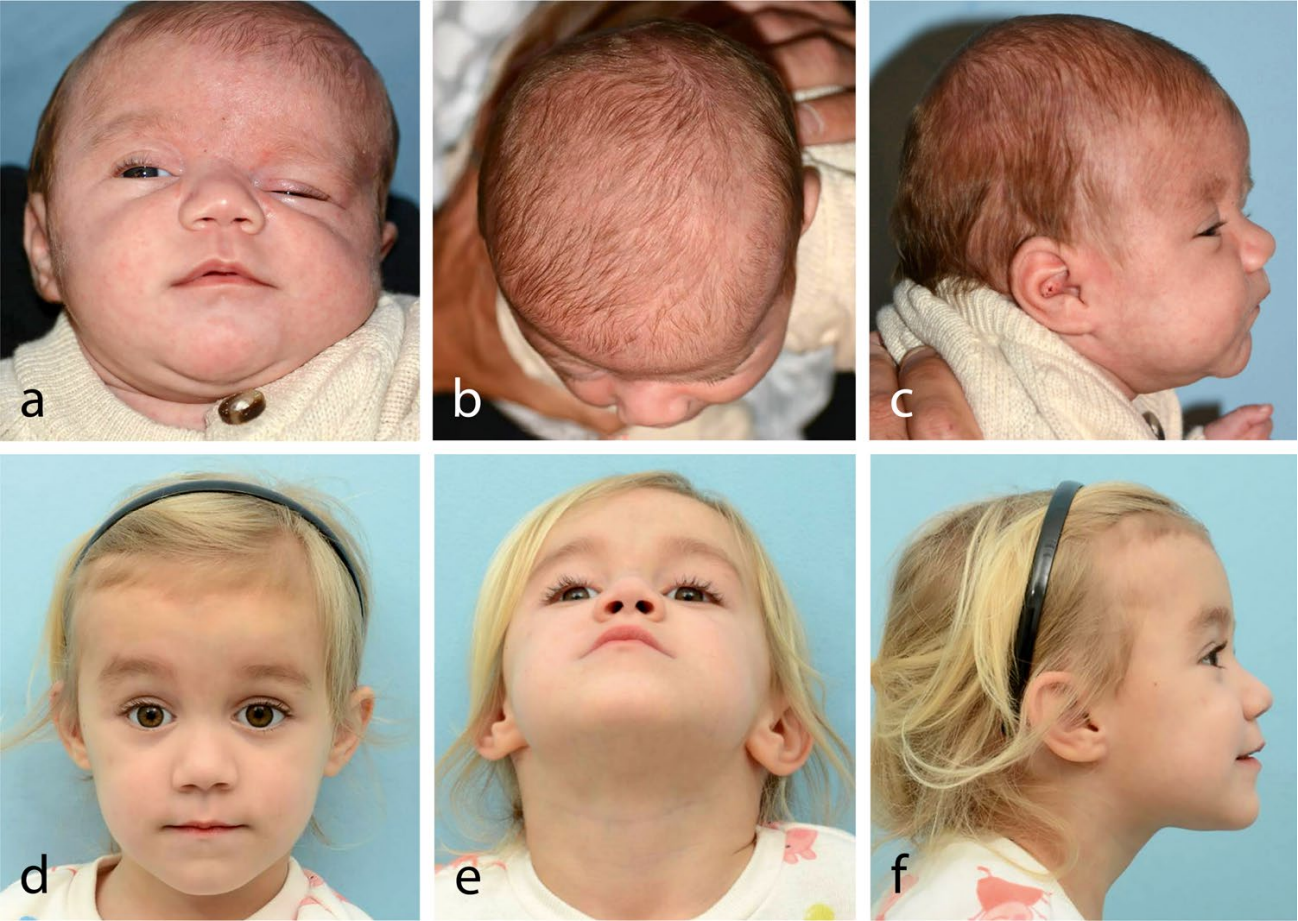
A female patient with right unicoronal synostosis is shown before undergoing endoscopic-assisted fronto-orbital distraction osteogenesis at five months of age (*top row*) and three years postoperatively (*bottom row*)

With regard to soft tissue measurements, all preoperative measurements except for palpebral width were likewise similar between groups. At nearly two years postoperatively, endo-FODO was associated with significant mean improvements in MRD1 symmetry ratio of 52.2, palpebral height symmetry ratio of 41.2, canthal tilt symmetry of 3.8°, and soft-tissue ODA of 4.4°. These postoperative changes are clinically meaningful, as MRD1 is used to identify blepharoptosis and upper lid retraction [40,41] while canthal tilt influences perceptions youthfulness and attractiveness [42,43]. Importantly, the improved symmetry in these parameters supports the critical relief of orbital dystopia in these patients **(**Fig. 4**)**. The anterior and inferior vector of distraction applies downward pressure on the facial skeleton and skull base to vertically align the orbits [35] while medialization of the orbital roof apex results from rotation of the frontal bone segment towards the contralateral side. [44]

**Fig. 5:**
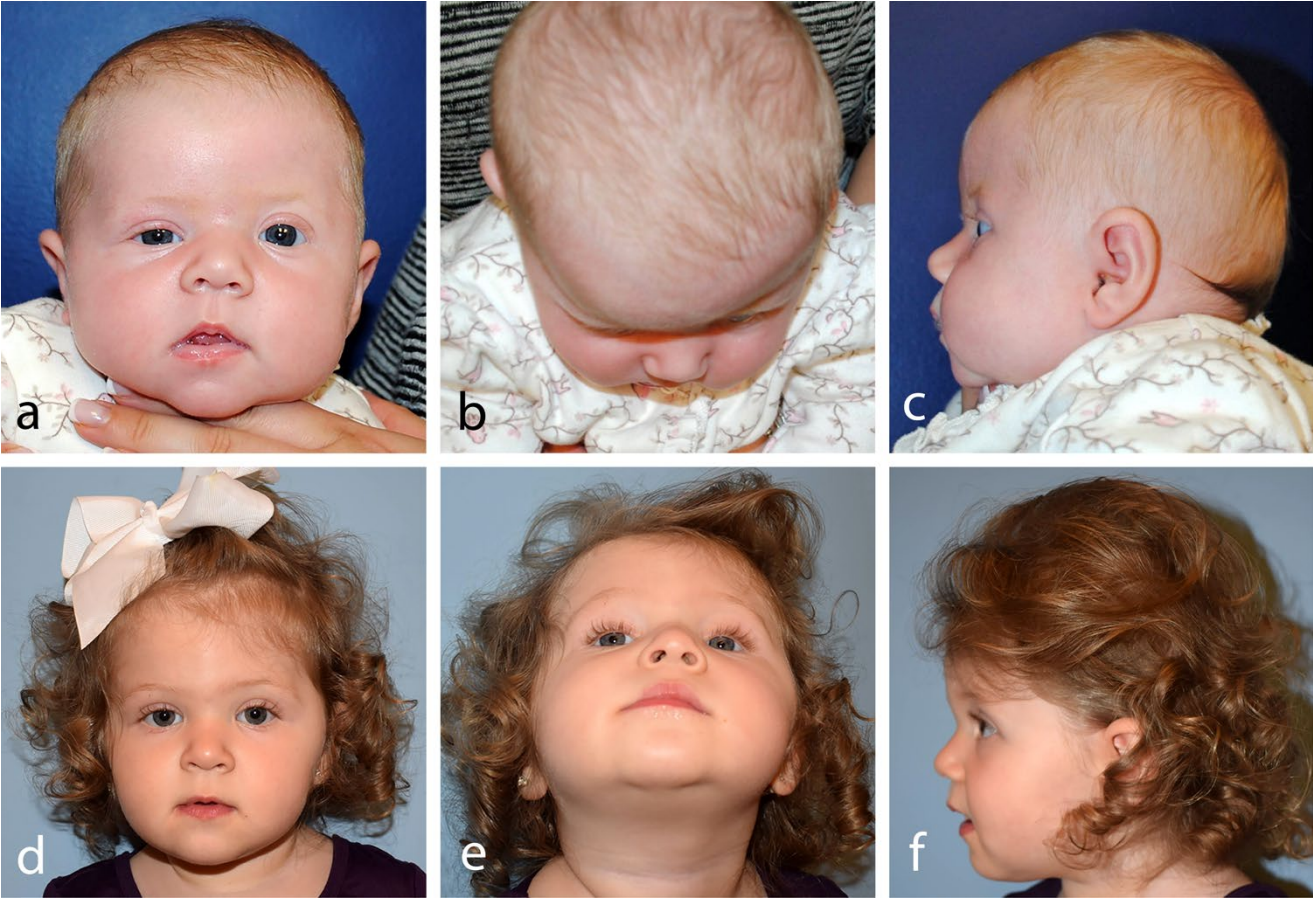
A female patient with left unicoronal synostosis is shown before undergoing open fronto-orbital distraction osteogenesis at four months of age (*top row*) and at two years postoperatively (*bottom row*)

Of greatest interest, these soft tissue changes after endo-FODO were not significantly different from those observed after open FODO (Fig. 5), which demonstrated significant mean improvements in MRD1 symmetry ratio of 29.5, palpebral height symmetry ratio of 14.6, and soft-tissue ODA of 3.8°. While open FODO was associated with a mean improvement in canthal tilt symmetry of 2.9°, this finding was not statistically significant as it was for endo-FODO. However, we refrain from drawing definitive conclusions regarding superior correction of canthal tilt given our relatively small sample size. Taken together, these aesthetic outcomes are encouraging as, despite similar osteotomy patterns and distraction protocols used, direct visualization is made more difficult through smaller incisions in endo-FODO. As postoperative ODA remained greater than zero in both treatment groups, undercorrection is possible. Interestingly, our recent analysis of long-term aesthetic outcomes after FODO versus FOAR found that soft-tissue ODA improved from a mean of 7.3° preoperatively to 1.7° at 7 years postoperatively, which could represent some normalization with growth overtime [45]. Nonetheless, slightly more overcorrection of the frontal deformity via increased distraction distances may be desirable to improve the durability of aesthetic outcomes.

Overall, there has been growing literature on morphometric outcomes of open fronto-orbital distraction for UCS correction [24,25,28,35,44]. A recent study at Asan Medical Center found that midface angulation decreased by an average of 4° at nearly four years postoperatively from open FODO. [46] Likewise, the Gothenburg group demonstrated that their method of fronto-orbital distraction by means of a coronal incision relieved orbital dystopia by lowering the ipsilateral orbit and straightening the nose and corrected deviation of the anterior cranial fossa in all patients at the time of distractor removal [35]. Future long-term investigations of morphometric outcomes as well as studies correlating skeletal and soft tissue changes after FODO would be of great value.

### Technique considerations

The potential advantages of FODO over FOAR have been previously described and include theearlier age at operation, larger and more gradual expansion, and preservation of the frontal bone flap’s dural attachment, which avoids the creation of a dead space after FOAR that may promote infection or postoperative relapse [22,23]. However, open FODO still relies on a traditional coronal incision. While this incision provides safe skull access, it remains a source of blood loss and appearance-related concerns among parents. Furthermore, scar widening occurs with age [47]. Endo-FODO minimizes this potentially stigmatizing scalp scar, and our recent assessment of cutaneous scarring at one year postoperatively using the Scar Cosmesis Assessment and Rating (SCAR) scale [48] demonstrated favorable scar cosmesis after endo versus open FODO for patients with UCS (1.00 vs. 1.83, *p* = 0.031) [49]. Nonetheless, the pterional and anterior fontanelle incisions may be extended bicoronally, if necessary.

While endo-FODO employs similar osteotomies as open FODO, a minimal-incision approach is more surgically challenging. Here we highlight several technical points that optimize safety and efficacy without prolonging operative duration. The pterional window, which obviates the resection of a large bony block at the temporal region, provides the critical working port for the most challenging osteotomy at the sphenoid wing and orbital roof **(**Fig. 1**)**. [30,49] This roughly 2-cm burr hole is ideally created over the junction of the frontal lobe, temporal lobe, and sphenoid wing. Difficulties can be encountered with variations in fronto-sphenoidal anatomy, which may complicate intracranial dissection and necessitate a slightly larger burr hole. Once the frontal bone, pterional, and lateral orbital osteotomies are performed, the ipsilateral frontal bone is carefully lifted to expose the sphenoid wing (Fig. 1). Two malleable retractors may be inserted through the coronal osteotomy to protect the frontal and temporal lobes, while a third malleable retractor is inserted through the blepharoplasty incision to protect the orbital contents as the orbital roof osteotomies are performed (Fig. 1). As viewing the tip of the blade or osteotome can be difficult when approaching the orbital roof intracranially, an endoscope can be inserted downward through the anterior fontanelle incision for visualization from above or upward through the lateral blepharoplasty incision for visualization from below. With an experienced craniofacial and neurosurgical team and increased familiarity with these principles, a safe operation resulting in effective fronto-orbital correction can be achieved.

### Study limitations

Limitations to this study exist, including its relatively small sample size. Most notably, we do not routinely collect postoperative CT scans for patients with UCS, limiting our ability to assess three-dimensional craniometric changes. However, we find it difficult to justify radiation exposure in these young, healthy patients, and existing craniometric outcomes studies on this topic do not assess soft tissue morphology as done in this study [26,46,50], which is important in shaping family satisfaction as well as perceptions of character attributes.^51^ At the same time, photogrammetric measurements are challenging to standardize, and we acknowledge the inherent variations in photo quality and visual interpretation using this methodology. Future studies should consider including several observers to improve reproducibility of the same measurements. As our experience with endo-FODO grows, we hope to increase sample size while following our current cohort to cranial maturity.

## Conclusions

Endo-FODO and open FODO are associated with favorable short-term aesthetic results, with both techniques demonstrating significant improvements in soft tissue periorbital symmetry and relief of orbital dystopia. Considering the decreased cutaneous scarring and perioperative morbidity associated with endo-FODO, our photogrammetric outcomes may tip the risk-benefit ratio towards supporting the adoption of a minimally invasive alternative to fronto-orbital distraction. Continued follow-up to cranial maturity is needed to evaluate the durability of aesthetic gains after endo-FODO.

## Data Availability

Data is provided upon request from the corresponding author.
